# Augmented Reality to Compensate for Navigation Inaccuracies

**DOI:** 10.3390/s22249591

**Published:** 2022-12-07

**Authors:** Miriam H. A. Bopp, Felix Corr, Benjamin Saß, Mirza Pojskic, André Kemmling, Christopher Nimsky

**Affiliations:** 1 Department of Neurosurgery, University of Marburg, Baldingerstrasse, 35043 Marburg, Germany; 2 Center for Mind, Brain and Behavior (CMBB), 35043 Marburg, Germany; 3 EDU Institute of Higher Education, Villa Bighi, Chaplain’s House, KKR 1320 Kalkara, Malta; 4 Department of Neuroradiology, University of Marburg, Baldingerstrasse, 35043 Marburg, Germany

**Keywords:** microscope-based navigation, augmented reality, AR, head-up display, navigation accuracy, brain shift, navigation update, spatial realignment

## Abstract

This study aims to report on the capability of microscope-based augmented reality (AR) to evaluate registration and navigation accuracy with extracranial and intracranial landmarks and to elaborate on its opportunities and obstacles in compensation for navigation inaccuracies. In a consecutive single surgeon series of 293 patients, automatic intraoperative computed tomography-based registration was performed delivering a high initial registration accuracy with a mean target registration error of 0.84 ± 0.36 mm. Navigation accuracy is evaluated by overlaying a maximum intensity projection or pre-segmented object outlines within the recent focal plane onto the in situ patient anatomy and compensated for by translational and/or rotational in-plane transformations. Using bony landmarks (85 cases), there was two cases where a mismatch was seen. Cortical vascular structures (242 cases) showed a mismatch in 43 cases and cortex representations (40 cases) revealed two inaccurate cases. In all cases, with detected misalignment, a successful spatial compensation was performed (mean correction: bone (6.27 ± 7.31 mm), vascular (3.00 ± 1.93 mm, 0.38° ± 1.06°), and cortex (5.31 ± 1.57 mm, 1.75° ± 2.47°)) increasing navigation accuracy. AR support allows for intermediate and straightforward monitoring of accuracy, enables compensation of spatial misalignments, and thereby provides additional safety by increasing overall accuracy.

## 1. Introduction

The introduction of image-guided surgery and neuronavigation in the 1990s had a great impact on neurosurgical practice and is nowadays an indispensable tool for a broad variety of cranial and spinal neurosurgical procedures [1,2]. Proving its clinical usefulness in the identification of e.g., deep-seated lesions, defining resection margins, or assisting in the preservation of functional risk structures, image-guided surgery and neuronavigation have become an intrinsic part of the surgical procedure itself [3,4].

The application safety and the potential advantages of image-guided surgery and neuronavigation essentially depend on the precision and accuracy of linking image space and physical space and thereby transferring the preoperatively obtained data onto the intraoperative surgical situs [2,4,5]. The term accuracy is not defined consistently in the current literature. According to [5] overall or clinical accuracy which is most relevant to the surgeon is a mixture of application accuracy and intraoperative accuracy. Application accuracy can thereby be divided into imaging accuracy, technical accuracy, and registration accuracy [5]. Imaging accuracy depends on the imaging modality (e.g., computed tomography (CT) imaging shows less geometric distortions compared to magnetic resonance imaging (MRI) [6]), and imaging parameters, such as resolution and slice thickness [7]. Due to less geometric distortions using CT data for patient registration leads to higher spatial patient-to-image registration accuracies. However, in brain tumor surgery, MRI is the modality of choice. Therefore, if not used for registration, at least during preoperative planning CT/MRI image fusion is performed. Geometric distortions present in MRI data lead to local suboptimal image fusion, providing additional spatial inaccuracies [8]. Technical accuracy of the navigation system itself depends on, e.g., the used tracking technology such as optical or electromagnetic [9,10], and is nowadays defined as being less than 3 mm in frameless stereotaxy [2,11,12]. Registration accuracy mainly influences application accuracy [13,14]. A variety of approaches are available, such as paired point matching of anatomical landmarks, bone screws, adhesive skin fiducials, laser surface matching, or automated approaches [3]. Fiducial registration with reported target registration errors (TRE) of about 1.8 to 5.0 mm [3,15] or surface matching techniques with even worse mean TRE of 5.3 mm [13] as noninvasive techniques are most commonly used. The introduction of intraoperative MRI or CT imaging with automated registration procedures significantly reduced the initial mean TRE being less than 1 mm [1,14,16,17,18,19]. Intraoperative accuracy is impaired significantly by brain deformations in the course of surgery due to, e.g., swelling, ongoing tumor mass reduction, the loss of cerebrospinal fluid, insertion of brain retractors, and effects of gravity after craniotomy and dural incision [4,19,20,21]. Furthermore, other intraoperative factors, such as draping, skin incision, duration of surgery, and changes in the spatial relation of head and reference array contribute to overall accuracy [2,3,4].

Even though the application accuracy can be improved at different levels to allow for an optimized initial patient-to-image registration, accuracy is constantly decreasing throughout the surgical procedure and in the worst case might even lead to an unacceptable mismatch [3,4]. To overcome alterations in the spatial relationship of the patient’s head and the reference array, most commercially available navigation systems enable an intraoperative landmark-based registration update. Therefore, readily available and uniquely identifiable landmarks, such as small drilled holes in the skull around the planned craniotomy, are acquired and can at any time be used to evaluate navigation accuracy in relation to those rigid bony structures and to restore accuracy if a discrepancy is seen by paired-point registration [22]. However, loss of accuracy up to the point of acquisition (e.g., draping, skin incision, interchange of reference arrays) cannot be compensated for in this way, the same accounts for the effects of brain shift. Brain shift can currently be addressed by intraoperative imaging (e.g., MRI, ultrasound (US)) if applicable, updating neuronavigation repetitively and partially allowing for a non-linear transfer of preoperative onto the intraoperative image data [20,23]. However, the usage of iMRI and especially repetitive iMRI is limited due to structural requirements, time consumption, restricted availability, and high costs [24]. In contrast, iUS can be performed at any time during surgery without significant interruption, is widely available, straightforward, cost-effective, and widely integrated in neuronavigation set-ups, but becomes more challenging in the case of lesions, where tumor outlines are not well defined in US data [25].

Another option also to account for smaller intracranial deformations is the application of augmented reality (AR) using the head-up display (HUD) of the operating microscope as suggested by [26], being complementary to existing approaches or in their absence being a useful alternative to obtain high navigation accuracy.

Augmented reality encompasses merging data from real-world environments and virtual information and vice versa. Within the surgical application, AR superimposes virtual information such as image data and additional information into the surgeon’s view of the patient. AR thereby complements and integrates the idea of standard surgical navigation relying on virtual reality only, providing a real-time updated 3D virtual model including anatomically relevant information overlaid on the surgical field [27]. First suggested by Kelly et al. and Roberts et al. in the 1980s, superimposing tumor outlines derived from preoperative CT data into the view of the surgical microscope, the commercialization of HUD microscopes in the 1990s introduced microscope-based AR to the broad neurosurgical community and has found its way into clinical practice [16,17,26,28,29]. In addition to visualizing multimodal fused image sets and outlined objects of interest, such as tumor or risk structures, on a navigation screen close to the surgical field, those objects can be visualized using the integrated AR display by superimposing the 3D objects in the operating microscope by the integrated HUD, supporting the surgeon’s mental transfer of relevant information between image space and surgical field and thereby reducing the demand for attention shifts and increasing surgeon comfort [17,27,30,31]. Thus far, AR-support in microscope-based neuronavigation was mainly used as intraoperative guidance tool. However, the concept of using AR for accuracy checks has been brought up in different studies investigating phantom data or small case series, most often focusing on bony structures, areas with limited brain shift or in spine surgery [32,33,34]. Using AR for evaluation of registration accuracy and improving registration accuracy throughout cranial surgery was recently suggested [4,26]. As proposed by [4], the cortical surface representation or prominent vascular structures, clearly identifiable intraoperatively, might be suitable to identify and compensate for inaccuracies prior to resection. The availability of the tool has been reported in small cohorts in case of skull base lesions [35] or aneurysm surgery [16].

The potential of AR-based registration evaluation and AR-based navigation update is not systematically evaluated in a large cohort of patients with intracranial lesions As high navigational accuracy is a critical prerequisite in navigation-supported neurosurgical applications, defining resection margins, or assisting in the preservation of functional risk structures, the aim of the present study was therefore to report on the capabilities of AR-support to evaluate registration accuracy and the opportunities and obstacles in using AR-support to compensate for registration inaccuracies. In this way, AR-based registration contributes to preserving high navigation accuracy throughout the procedure, complementary to alternative approaches, but also to obtaining and gaining high accuracy in the absence of methodological alternatives, e.g., due to missing intraoperative imaging modalities.

## 2. Materials and Methods

### 2.1. Study Cohort

Data of 293 consecutive patients (male/female: 152/141, mean age: 58.00 ± 14.69 years), who underwent neuro-navigated microsurgical resection of suspected lesions (single surgeon study, C.N.) between December 2018 and September 2022 were analyzed within this study. All included patients provided written informed consent before participation. An ethics approval for prospectively archiving and collecting routine clinical and technical data was obtained in accordance with the Declaration of Helsinki and approved by the local ethics committee at the University of Marburg (Study 99/18).

### 2.2. General Set-Up and Automatic Patient Registration Using AIRO iCT

For automatic patient registration, a 32-slice movable CT scanner (AIRO, Brainlab, Munich, Germany) was used, which is immediately embedded in the navigation setup, consisting of a double monitor ceiling-mounted navigation system (until June 2022) or since July 2022 a mobile single monitor navigation system (Curve CM and Curve Navigation, Brainlab, Munich, Germany), paired with a dual-display in-wall system (Buzz, Brainlab, Munich, Germany) to allow for an in-parallel view of different applications of the navigational setup (e.g., navigation screen, microscope view). In all cases an axial low-dose scanning protocol (7.1 mA, 120 kV, scan length 6.2 cm, resulting dose-length-product 17.8 mGy*cm) was used without weight modulation, exposure time of 1.92 s and 33.92 mm table movement per rotation, fixed slice collimation of 1.06 mm, a reconstructed slice thickness of 1 mm and matrix size of 512 × 512.

Under general anesthesia, the patient’s head is fixated within a radiolucent carbon head clamp (DORO) using standard metallic head fixation pins. While related artifacts are not immediately relevant in the low-dose registration scan but should not cover the area of interest in the case of a local follow-up high-dose control scan during surgery, the pins are placed outside this area of interest. A radiolucent patient reference array is attached on the left side of the head clamp.

In some of the patients, the automatic registration scan was performed at this stage of surgery. To evaluate a target registration error (TRE) to measure all-over registration accuracy before surgery, three adhesive skin markers are attached that are not used for registration. Then, the operating table is rotated 90°. The navigation camera is adjusted in a way that there is a clear line of sight of the patient reference array’s geometry and the reflective markers attached to the iCT scanner. The initial low-dose iCT scan is performed without a prior localization scan with a scan length of 6.2 cm (measured manually) covering the skull base. Afterward, the patient table is rotated back, images are automatically transferred to the navigation system allowing for automatic patient registration. Registration accuracy is performed using the applied three adhesive skin markers attached within the scan area. Placing the tip of the navigation pointer in the divot of each fiducial the TRE can be calculated as the offset between the tip of the pointer in the divot of the skin marker and the divot of the marker within the image data. After verification of patient registration, skin disinfection and draping are performed. Thereby, the upper part of the non-sterile patient registration array is interchanged with a sterile one. In parallel, the preoperative surgical plan is co-registered rigidly with the low-dose iCT registration scan; this way, navigation can be applied immediately.

Alternatively, the automatic registration procedure can also be performed later in the course of surgery, e.g., in cases where navigation is not needed due to prior craniotomy (tumor recurrent) or standard approaches. In those cases, after head fixation, skin disinfection and draping are performed. After skin incision and craniotomy or preparation of the bone flap, at this stage, a registration scan can be performed. Therefore, the skin flap is moved back to cover the surgical site. The upper part of the sterile patient registration array is interchanged with a non-sterile one after additionally draping the sterile field. Then, the operating table is rotated 90°, the navigation camera is adjusted properly and a low-dose iCT registration scan is performed after a lateral scout scan (10 mA, 120 kV) to adjust the scan range (6.2 cm). Afterward, analogously the OR table is rotated back, and images are automatically transferred for patient registration. While performing rigid co-registration with the prepared preoperative surgical plan, the upper part of the non-sterile patient reference array is interchanged again with the sterile one after removing the additional drape.

**Figure 1:**
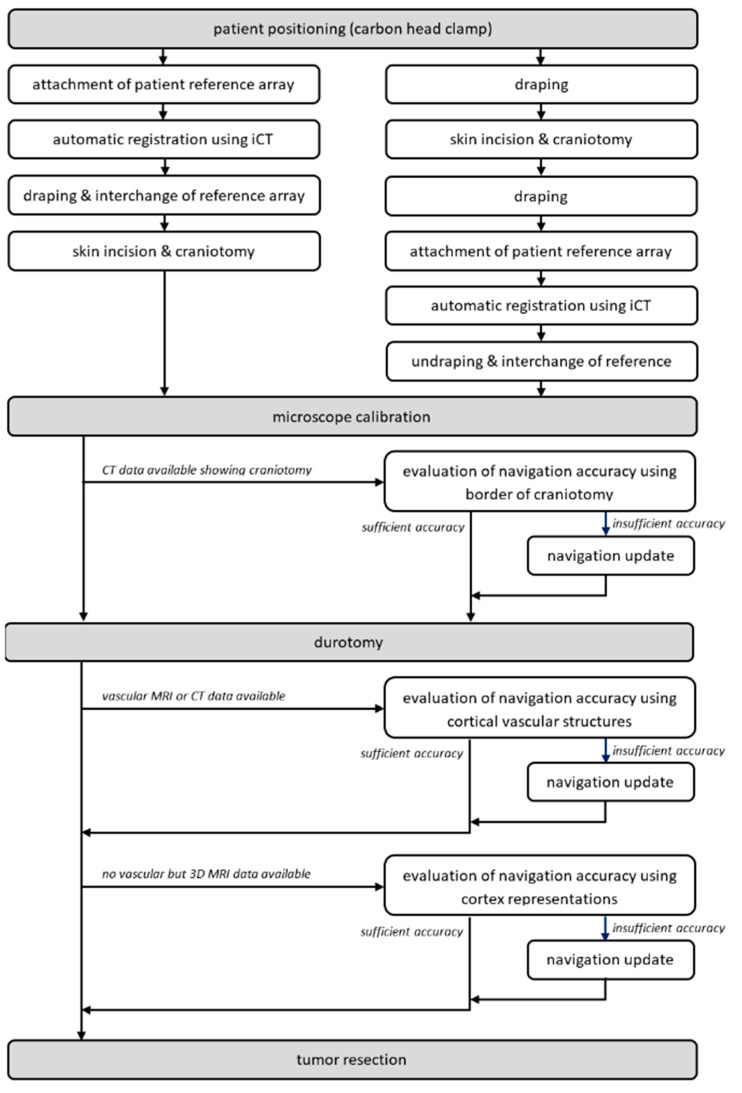
Overall workflow incorporating the application of automatic registration using iCT (prior to or after craniotomy), microscope calibration, AR-based navigation verification, and a potential update prior to the durotomy using bony landmarks and AR-based navigation verification, and a potential update using vascular structures and/or cortex representations depending on data availability.

After the craniotomy and before the durotomy, typically navigated ultrasound is used, allowing for an immediate evaluation of navigation accuracy and acquisition of 3D ultrasound data sets for navigation purposes, see [21,25]. After the durotomy, the microscope is initialized and used for microscope-based navigation. For further details on the workflow, see [1,14]. To facilitate AR-support throughout surgery, head-up displays (HUD) integrated into the operating microscopes, Pentero/Pentero 900/Kinevo 900 (Zeiss, Oberkochen, Germany), were used with no need for further AR-supporting devices. After calibration, AR-support is used to evaluate and, if necessary, to increase navigation accuracy investigating bony landmarks, cortical vascular structures, or cortex representations, for an overview of the workflow see Figure 1.

### 2.3. Calibration of the Operating Microscope and Augmented Reality

Integrated into the navigation set-up and providing AR-support throughout surgery, the operating microscope was tracked using an attached microscope registration array that can be aligned in two different ways (pointing to the front and pointing to the right side). Calibration of the head-up display visualization of the used operating microscope (Pentero/Pentero 900/Kinevo 900, Zeiss, Oberkochen, Germany) was obtained using the Microscope Navigation Element (Brainlab Elements, Brainlab, Munich, Germany) that automatically detects the geometry of the attached four spherical marker geometry, estimates the position of the central divot, and the calibration marks provided on each of the reference array’s arms, enabling an AR visualization of the reference array. By focusing on the reference array’s central divot and manually translating the displayed crosshair and visualized AR outlines to match the outline of the reference array and displayed microscope’s crosshair. Analogous adaptions can be made by focusing on the calibration marks. Thus, the AR can be used to achieve an optimal matching and referencing of the surgical microscope to minimize navigation inaccuracies due to reduced AR accuracy (intrinsic parameters, miscalibrated HUD).

**Figure 2:**
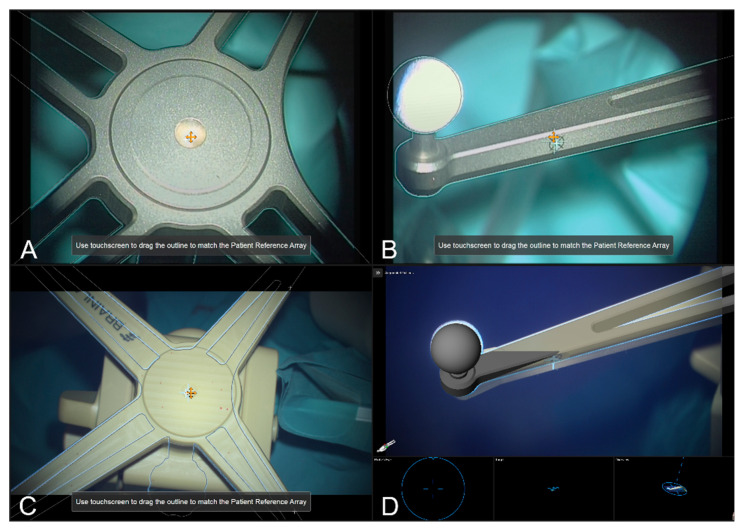
The AR representation of the cranial patient reference array visualizing matching the standard stainless-steel reference unit (**A**,**B**). The 2D and 3D AR representations overlaid onto the radiolucent cranial patient reference array used within the iCT set-up matching the central divot (**C**), and the spherical marker and calibration mark along the reference array’s arm (**D**).

In the microscope application, thus far only the stainless-steel cranial reference array is available as the corresponding AR visualization perfectly matches the real reference array if matched correctly. In the case of using intraoperative CT imaging as done in this study, there is a discrepancy seen in the 3D AR visualization as the specific layout is not yet provided by the software, but the integrated markers (e.g., divot), and the reflecting spherical markers, can be used to verify the matching of AR and reality, see Figure 2.

**Figure 3:**
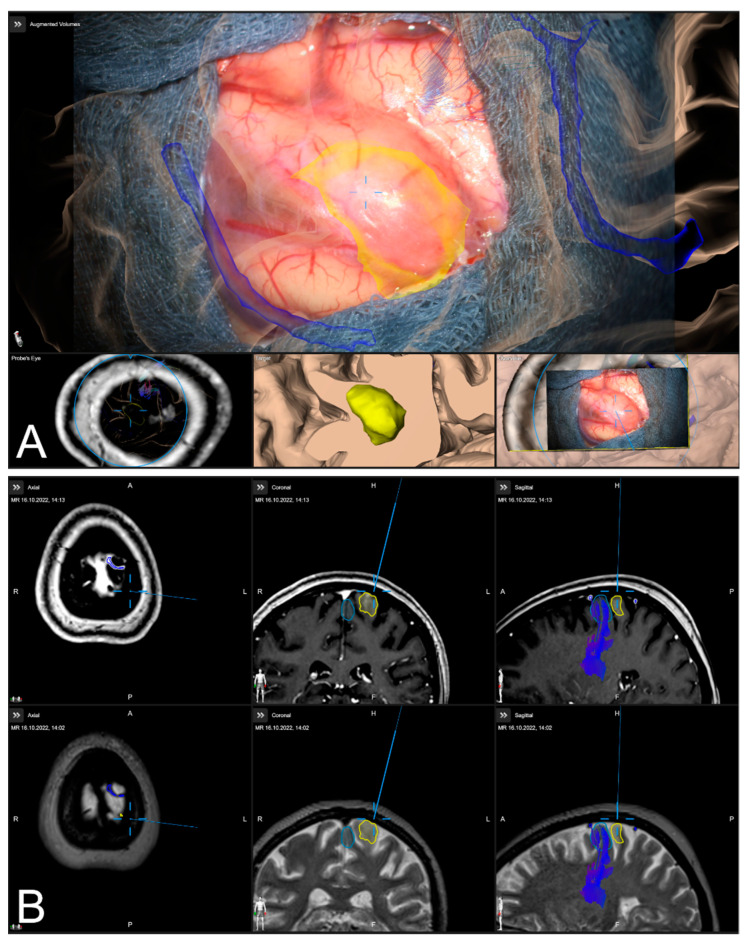
After calibration of the microscope, besides HUD-based visualization within the microscope, the Microscope Navigation Element (**A**) and Cranial Navigation element (**B**) are displayed on monitors close to the surgical field. The microscope application allows for a visualization of outlined objects (yellow: tumor, blue: precentral gyrus, corticospinal tract) superimposed on the microscope video (**A**, top), or probe’s eye view, target view or outlined 3D anatomy in relation to the microscope video frame (**A**, bottom, left to right). In parallel, in the navigation application multimodal fused image sets with outlined structures are visualized in, e.g., axial, coronal, and sagittal view (**B**).

After calibration of the surgical microscope, AR-support including all pre-segmented objects is available. Outlined objects, such as the tumor or vascular or functional risk structures, can then be visualized using the AR display by superimposing the 3D objects in the operating microscope by the integrated HUDs. In parallel, multimodal fused image sets are visualized in the Cranial Navigation Element (Brainlab, Munich, Germany) on a monitor close to the surgical field. In addition, within the Microscope Navigation Element (Brainlab, Munich, Germany) objects can be displayed, e.g., superimposed on the microscope video or within a probe’s eye view of the registered image data. Alternatively, the microscope video can be superimposed on a 3D visualization of the patient data including all objects and relevant pre-segmented structures intuitively relating the microscope video frame and 3D anatomy (see Figure 3).

### 2.4. Microscope- and AR-Based Evaluation of Navigation Inaccuracies and Navigation Update

Despite high navigation accuracy achieved by applying an automatic patient registration approach utilizing low-dose iCT registration scan overcoming navigational errors of fiducial-based or surface-based approaches [1,14,16,17,18,19], during surgery navigation inaccuracies might occur. However, various aspects might lead to decreased accuracy. In addition to the movements of the patient itself, due to possible too-low sedation or shifting of the patient’s head in the head clamp, the handling of the patient reference array during draping and surgery might cause inaccuracies [2,3,4]. In addition to those mechanical aspects, obvious changes in anatomy due to brain shift and/or ongoing tumor resection lead to discrepancies between the real-world anatomy and image data that was acquired before surgery. As brain shift itself is typically addressed using intraoperative imaging techniques, such as ultrasound, computed tomography, or magnetic resonance imaging [3,20,23], estimating the amount of brain shift or updating intraoperative navigation, the microscope application (Brainlab Elements, Brainlab, Munich, Germany) allows compensating for inaccuracies by adjusting the image set and in situ patient anatomy within the recent microscope’s focal plane. The navigation update feature offers two different possibilities for alignment. On the one hand, a maximum intensity projection (MIP) of the image set at the level of the current focus plane can be used. On the other hand, the 3D outlines of the pre-segmented objects can be displayed according to their recent registration. If a discrepancy is seen the visualized MIP or object representation can be transformed by shifting and rotating the overlay within the focus plane to match the patient’s anatomy. After verification of the altered correlation, the updated registration will be applied to overcome the discrepancies at this level.

#### 2.4.1. Verification of Navigation Accuracy Using Bony Landmarks

**Figure 4:**
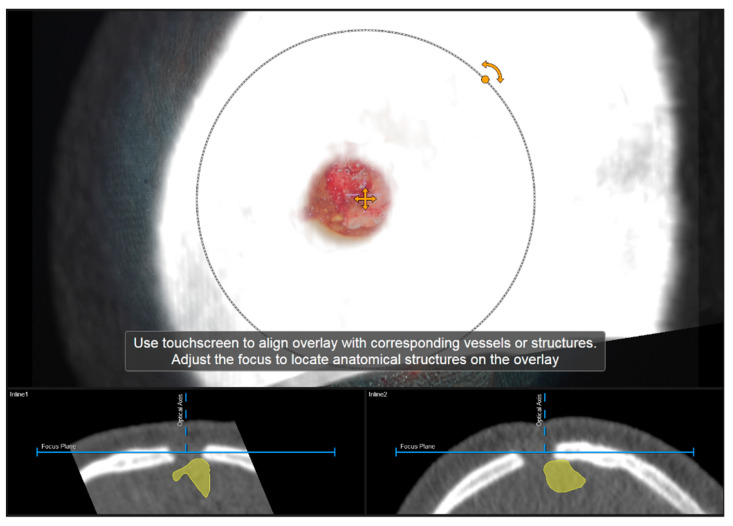
Evaluation of navigation accuracy using a CT-based MIP projection centered at a burr-hole of a prior biopsy surgery, showing a good match of image and patient data (In parallel view of MIP projection (upper part) and inline views with the recent focus plane (blue line) and the optical axis (dashed blue line) in the bottom part).

Navigation accuracy can be evaluated using bony landmarks. In cases the iCT registration scan was performed after craniotomy or in cases of recurrent tumors or patients with prior burr-hole surgery (e.g., biopsy) preoperative CT imaging-based navigation accuracy can be evaluated using a CT-based MIP or even outlines of the segmented bone flap. Focusing on the edge of the craniotomy at different areas (if available within the data) or using a burr-hole (see Figure 4) or bony structure of interest to match image content and patient anatomy thereby allows for a visual comparison of image content or segmentation of a patient’s anatomy. By either verifying navigation accuracy or even adjusting the image sets to the recent in-focus plane anatomy of the patient by translating and/or rotating the image data within the recently visualized plane, navigation inaccuracies can be compensated for according to in-plane errors. If corrected, the new registration needs to be verified by the user using the pointer to also account for registration applicability beyond the scope of the in-plane update.

#### 2.4.2. Verification of Navigation Accuracy Using Cortical Vessels

Navigation accuracy can also be evaluated using cortical vessels. After durotomy, the apparent cortical vessel structures can be used to investigate the accuracy, either verifying accuracy or trying to compensate for inaccuracies. A MIP representation is thereby created by image data ideally clearly picturing vessels, such as CT angiography data (CTA), T1 contrast-enhanced MRI data (T1-CE), or MR time-of-flight angiography data (ToF). Focusing on prominent cortical vessels, especially close to branching, within the surgical site the corresponding MIP representation of vessel structures is visualized, and navigational accuracy can be evaluated. If a mismatch is seen within the recent focus plane, those inaccuracies can be compensated again by a rigid transformation of the image data. In case a proper vessel segmentation is available besides the MIP representation also the object representation can be used. Especially in cases where no proper data for a MIP-based vessel representation is available a manual segmentation of cortical vascular structures within, e.g., T2 weighted MRI data can be utilized. If corrected, the new registration that is applied for further navigation needs to be verified as described above.

#### 2.4.3. Verification of Navigation Accuracy Using a Cortex Segmentation

In addition to the previous navigational accuracy evaluation based on bony or vascular landmarks, a segmentation of the cortical profile can be investigated. Especially when no bony imaging landmarks are available or in cases with no sufficient imaging data for outlining and visualizing vascular cortical structures is present, the visualization of the cortical profile can be a helpful tool. Therefore, during planning an object representation of the cerebrum can be created using the automatic segmentation algorithm (Object Manipulation, Brainlab Elements, Brainlab, Munich, Germany). In those cases, the object representation is used within the navigation update feature to match the cortical profile and the cortical object representation within the recent focus planes. Analogous to the evaluation procedure described above, potential in-plane inaccuracies can be compensated for by translation and/or rotation of the image content followed by a registration verification.

In addition to the 2D object representation available within the navigation update feature, in the case of the cortex being not as straightforward as in the case of bony or vascular structures, the microscope navigation provides different views to visualize the preoperative image data and the microscope video data during surgery. The Overview View enables the microscope video to be superimposed on a 3D visualization of the patient data including the pre-segmented objects and structures of interest and thereby related intuitively the video frame and the 3D anatomy. By moving the focus plane (superimposed microscope video) along the optical axis of the microscope, the match or mismatch of the cortical representation can be easily detected. In case of a mismatch, another navigation update needs to be performed.

### 2.5. Analysis of AR-Based Navigation Accuracy Evaluation and Update

In all cases with CT data depicting the area of craniotomy (preoperative CT data in patients with previous surgery or intraoperative CT data acquired after craniotomy incorporating this are within the limited scanning range), AR-based navigation accuracy is intraoperatively evaluated using the borders of craniotomy. Depending on the surgeon’s impression, the quality of matching is stated as “accurate” (good spatial overlap of MIP and patient anatomy) or “inaccurate” with the need to further align the image space by translation and/or rotation of the 2D MIP data.

In all cases (partially after evaluation of bony landmarks) with available vascular data (T1-CE, ToF, and CTA) AR-based navigation accuracy is intraoperatively evaluated at prominent cortical vascular structures. Analogous the surgeon intraoperatively decides on “accurate” or “inaccurate”, then followed by realignments, or in case of non-sufficient vascular data (image quality, contrast), on “non-sufficient data”.

In cases where the above-mentioned data is not available or sufficient, but a 3D MRI data set for cortex segmentation is accessible, this segmentation is used to evaluate navigation accuracy analogously, the surgeon intraoperatively classifies navigation accuracy in “accurate” or “inaccurate” followed by a navigation update. In addition, the video superimposed on a 3D visualization of the patient data including segmented objects can be used for verification.

If a navigation update was performed, the amount of translation and/or rotation is gained from the information provided by the navigation system’s logfiles afterwards.

## 3. Results

### 3.1. Patient Characteristics

In total, 293 patients (male/female: 152/141, mean age: 58.00 ± 14.69 years) were included in the study. Additionally, 67 out of 293 patients (22.87%) had previous surgery. All patients were included and underwent surgery due to suspected lesions or recurrence and underwent AR-supported microscope-based resection. Neuropathological diagnosis revealed glioma (n = 156), metastasis (n = 53), meningioma (n = 31), cavernoma (n = 11), reactive altered tissue after radiation therapy (n = 10), reactive altered tissue n = 4), craniopharyngioma (n = 3) or other lesions (n = 22).

### 3.2. Automatic Patient Registration

All patients underwent surgery utilizing automated patient registration with intraoperative CT imaging. In 166 out of 293 cases (56.66%), initial patient registration using iCT was performed before the skin incision. In the remaining cases (n = 127, 43.34%), iCT for automatic patient registration was performed after craniotomy (e.g., due to preexisting craniotomy in patients with tumor recurrence or a standard approach with no need of navigation for skin incision and craniotomy). In cases with automated patient registration before skin incision, the mean TRE for the initial registration was 0.84 ± 0.36 mm (range: 0.17 to 2.52 mm) showing a high registration accuracy.

### 3.3. Verification and Update of Navigation Accuracy using the Operating Microscope and AR

In all cases, (n = 293) navigational accuracy was evaluated using the operating microscope and/or AR visualization before resection making use of bony landmarks, cortical vascular structures, and/or cortex representations, see Table 1.

**Figure 5:**
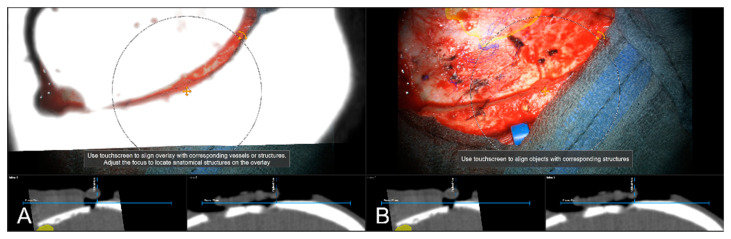
Using an intraoperatively conducted CT scan for automatic patient registration after craniotomy for evaluation of navigation accuracy showing a sufficient in-plane match of MIP (**A**) and patient anatomy (**B**). (In parallel view of MIP projection and patient anatomy (upper part) and inline views with the recent focus plane (blue line) and the optical axis (dashed blue line) in the bottom part).

**Figure 6:**
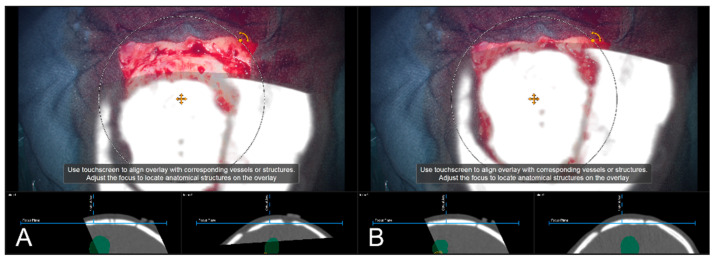
Navigation inaccuracy seen in the recent focus plane utilizing a MIP of the intraoperative automatic registration CT image set acquired after craniotomy showing the translational mismatch of MIP and patient anatomy (**A**) and the match of MIP and patients anatomy in the recent focus plane after manual correction (translation) of the visual misalignment (**B**). (In parallel view of MIP projection (upper part) and inline views with the recent focus plane (blue line) and the optical axis (dashed blue line) in the bottom part).

In 85 cases (29.01%), a pre-resectional CT data set (preoperative routine CT data or intraoperative CT conducted for automatic registration) was used to examine navigation accuracy using bony landmarks, such as burr-holes (one case, prior biopsy) and edges of craniotomy itself. In 83 cases (97.65%) out of those cases, navigation accuracy was evaluated to be sufficient, see Figure 5. In two cases (2.35%), there was a need for updating the used registration to account for the obvious in-plane mismatch of MIP and patient anatomy by rigidly translating the image data within the recent focus plane, see Figure 6. In both cases, the misalignment was compensated for by translating the projected data by means of 6.27 ± 7.31 mm.

**Figure 7:**
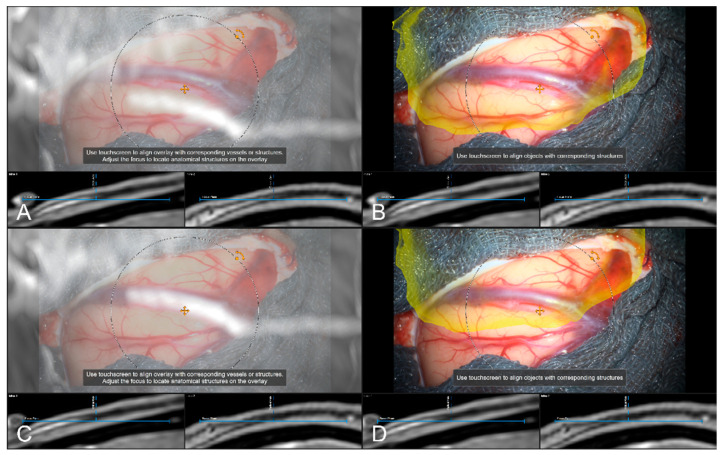
Navigation inaccuracy visualized in the recent focus plane utilizing a MIP of a preoperative T1-CE image showing the translational mismatch of MIP and patient anatomy (**A**,**B**) and the match of MIP and patients anatomy in the recent focus plane after manual correction (translation) of the visual misalignment (**C**,**D**). (In parallel view of MIP projection and patient anatomy (upper part) and inline views with the recent focus plane (blue line) and the optical axis (dashed blue line) in the bottom part).

**Figure 8:**
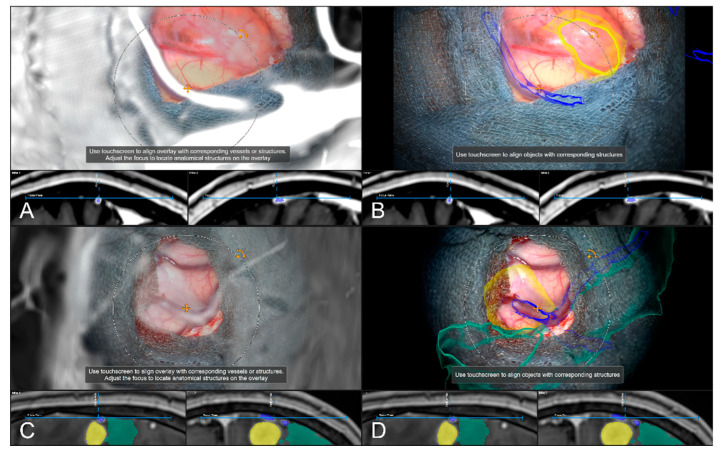
High spatial navigation accuracy seen in the recent focus plane utilizing a MIP of a preoperative T1-CE image (**A**,**C**), and patient anatomy enhanced with segmented vascular structures (blue), tumor outlines (yellow) and precentral gyrus (green) (**B**,**D**) in two patient cases, showing the different quality of preoperative imaging data (In parallel view of MIP projection and patient anatomy (upper part) and inline views with the recent focus plane (blue line) and the optical axis (dashed blue line) in the bottom part).

In 242 cases (82.59%), a vascular representation gained by available preoperative CTA, T1-CE, or ToF angiography data was used to evaluate navigation accuracy after dural incision, whereas in the remaining 18 cases (6.14%), no sufficient representation of cortical vascular structures could be obtained due to a lack of sufficient 3D data or poor contrast enhancement. In 43 out of those 242 cases (17.77%), a need to further realign the image data within the recent focus plane (see Figure 7) was seen, whereas in 181 cases (74.79%), navigation accuracy was determined to be sufficient (see Figure 8), partially due to prior evaluation utilizing bony landmarks. In all 43 cases, showing an inaccurate match of image and patient data, these local inaccuracies were successfully compensated for by rigid 2D transformation of the image data by translation on average of 3.00 ± 1.93 mm and a rotation on average of 0.38° ± 1.06°.

**Figure 9:**
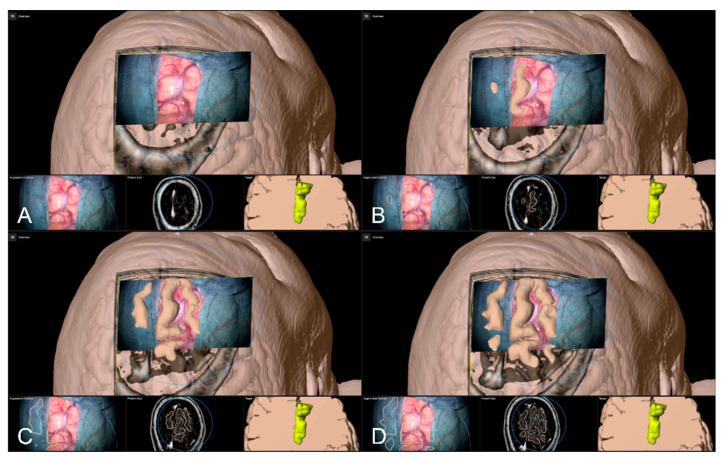
Superimposing the microscope video on the 3D visualization of patient MRI data including the pre-segmented objects (cerebrum and tumor) intuitively relating video frame and 3D anatomy (upper part in **A**–**D**), in parallel view of AR-supported microscope view, probe’s eye view and target view (bottom part in **A**–**D** from left to right). Moving the focus plane (superimposed microscope video) along the optical axis of the microscope (**A**–**D**), the registration quality can be evaluated showing a sufficient match in this case.

Alternative to bony and/or vascular landmarks, in 40 cases (13.65%) a cortex representation gained by automatic segmentation of cranial structures was used to evaluate and verify navigational accuracy. As the in-plane object representation is not straightforward, the Overview View was used firsthand in all cases to evaluate navigation accuracy allowing for superimposing the microscope video on the 3D visualization of the segmented data, see Figure 9. If a mismatch was seen, switching to the navigation update feature allowed for an in-plane transformation of the date to overcome the seen misalignment in the focus plane. This was the case in two patients (5.00%), whereas in the remaining 38 cases (95.00%), there was no need for further alignments was seen. In both cases, a linear transformation by on average 5.31 ± 1.57 mm and 1.75° ± 2.47° led to sufficient accuracy.

## 4. Discussion

Neuronavigation systems are expected to work with high accuracy throughout surgery, but there are several factors influencing navigation accuracy at different stages of the surgical procedure, in the worst case leading to an unacceptable mismatch between image and patient data and complete loss of neuronavigation capabilities [3,4,36].

With knowledge about a certain kind of decrease during surgery and about system limitations and achieved initial accuracy [37], there is a need for tools to verify patient-to-image registration at any time during surgery. AR-support using image injection techniques is currently widely used in neurosurgical operating rooms. Up to now mainly used as a guiding tool by using the microscope’s focal point as an integrated pointer. Integrating all relevant information in the surgical field of view and supporting the surgeon’s mental transfer of relevant information from image space to the surgical field, AR-support further reduces the demand for attention shifts and increases surgeon comfort [17,27,30,31]. In the same way, it can be exploited to evaluate registration accuracy during the approach but also consequently to improve registration accuracy [4,26].

In this study, AR-support and neuronavigation were used to evaluate navigation accuracy with bony landmarks (e.g., borders of craniotomy) in 85 cases, cortical vascular structures in 242 cases, or a 3D cortex representation in 40 cases. In two cases verifying accuracy with bony landmarks, a mismatch was seen that was sufficiently compensated by 2D translation. In all 43 cases with vascular structures for accuracy evaluation, a sufficient 2D realignment was performed within the focal plane of the microscope. In 18 cases, preoperative vascular data was not utilizable for accuracy verification. Especially for cases with larger craniotomy or non-availability of vascular image data, cortex representation was used. In 2 out of 40 cases a misalignment was seen, which was adequately compensated for by rigid alignments within the focus plane.

Even though there is no consistent definition of “accuracy” and contributing factors, the initial patient registration is known to be one of the main factors contributing to navigation accuracy [3,13,30,38]. Patient registration can typically be performed using a landmark- or surface-based registration approach. In landmark-based approaches, commonly adhesive skin markers (fiducials) are placed on the patient’s head prior to preoperative imaging (CT or MRI) [1,2]. Intraoperatively, after patient positioning and head fixation, a paired-point registration is performed. However, registration accuracy varies tremendously with a reported TRE of about 1.8 to 5.0 mm [3,11,15]. Thereby, the number, position, and spatial placement pattern of fiducials as well as skin shift during image acquisition (e.g., due to cushions used for head fixation), after intraoperative head fixation, and/or introduced by the navigated pointer during the user-dependent and error-prone registration procedure contribute to decreased accuracy [2,14,19,39,40,41]. Alternatively, surface-based approaches combining the use of anatomical landmarks and laser surface matching offer a somewhat clinically more practical opportunity for patient registration, as no additional imaging procedure with adhesive skin markers is required. Nevertheless, the registration accuracy is reported to be even worse compared to the fiducial-based approach and heavily depends on image modality and quality [13,37]. The introduction of intraoperative 3D imaging enables an automatic and user-independent registration procedure significantly increasing the initial registration accuracy with reported TRE being less than 1 mm [1,14,16,17,18,19,42]. In this way, the initial patient registration accuracy as one of the main factors contributing to overall clinical accuracy can be increased compared to user-dependent approaches.

Surprisingly, despite the high accuracy that was achieved by automated iCT-based registration, the evaluation of bony landmarks (borders of craniotomy), if available, showed a significant mismatch of image data and patient anatomy in two cases. This mismatch might be due to operational factors. When analyzing the intraoperative workflow for patient registration, the upper part of the sterile patient reference array interchanged with a non-sterile one after additional draping of the sterile field for iCT registration Afterwards, the non-sterile one is removed, and the additional drapes and the sterile one is attached to the bottom part of the reference array. In this way, e.g., the attachment of the upper part of the reference array, the folds of the drape between the bottom and upper part of the reference unit and the tension of the drape are forces that are applied to the reference unit interconnected to the head clamp and can contribute to those inaccuracies. This finding supports previous reports on the operational influence factor by handling the navigation equipment and the relevance of a constant spatial relation between the reference array and the patient’s head that might slightly change during surgery (shift between head and head clamp and/or head clamp and reference array) [2,3], and promotes the need for even pre-resectional intraoperative evaluation of accuracy for stable and high accuracy and additional safety throughout the procedure.

Previous studies report a constant decrease in navigation accuracy in the course of surgery, due to the attachment of surgical drapes, skin incision, skin retractors, trepanation, craniotomy, and even the duration of surgery [3,4,37]. Thus, this is mainly influenced the physical or patient-to-image registration accuracy before durotomy which is typically based on landmarks (skin, bone) or 3D surface representations. One approach to obtain high initial navigation accuracy would be to acquire intraoperative bony landmarks (e.g., small drilled holes around craniotomy) before significant errors occur to on the one hand verify accuracy, and on the other hand restore registration using a landmark-based approach in case of inaccuracies [22]. Changes in the spatial relation of the patient’s head and reference array that occur before the acquisition of intraoperative landmarks cannot be compensated for with this approach. In these cases, AR-based navigation updates might serve as an alternative or complementary approach using preexisting landmarks, if applicable. However, clinical accuracy is most relevant intracranial and should be monitored and accounted for similarly. Therefore, readily available and uniquely identifiable landmarks need to be investigated, such as prominent cortical vessels or the cortical profile [4]. The need for these accuracy checks and compensation for inaccuracies is of special interest as brain shift is an unavoidable source of error that reduces the value of neuronavigation without influencing its physical accuracy addressed before [3]. Whereas a slight shift of the brain within the cranium is dependent on the body and head position, after durotomy the brain might shift in a preoperatively more or less unpredictable amount and manner due to, e.g., increased intracranial pressure, cerebrospinal fluid outflow, effects of gravity, the insertion of brain spatula, and ongoing resection [2,3,4,20,43,44,45]. Various approaches have been developed to address this phenomenon, including intraoperative MRI [20,46,47,48] or ultrasound [23,49,50], or to use the intraoperative data to update planning and navigation, or to use non-linearly transform complex preoperative data onto the recent intraoperative situation. Thus, non-linear deformations of the brain itself or even a shift of a surgical target within the brain can be addressed. However, the application of iMRI, and especially repetitive iMRI, throughout the surgical procedure is limited due to the restricted availability, structural requirements, increased time consumption, and high costs, and might rather be used for resection control or navigation update in the late course of surgery. IUS can be performed without significant time consumption at any time during surgery, is cost-effective, and is widely integrated in the neuronavigation environment. However, a non-linear update of preoperative data and iUS data is not yet clinically available. User-based rigid image fusion alternatively might be limited in cases of lesions where tumor outlines are not well defined in US data. AR-based navigation update might serve as a valuable, fast, and straightforward tool to overcome initial deformations after durotomy, investigating prominent cortical vessels, or the cortical profile as shown here. The use of AR-based navigation verification and update using bony landmarks in the late course of surgery might be a helpful in case the spatial relationship of reference array and patient’s head changes but cannot compensate for intracranial alternations due to resection. The use of cortical structures and AR for verification and update in the late course of surgery might be limited due to ongoing resecting, severe shift due to the insertion of spatulas, and so on. Nevertheless, if appropriate (subcortical) intracranial landmarks are available, such as vascular structures as shown by [16], in the case of aneurysm surgery, this approach might also be applicable at this time during surgery, but needs to be investigated further.

In cases with small lesions and small craniotomies that require no major manipulations in the cortex, superficial monitoring and compensation of inaccuracies might be sufficient throughout the procedure [4]. Nevertheless, with knowledge about a continuous decrease in accuracy during the procedure and, therefore, a need for intermittent accuracy checks to assure high accuracy and additional safety, it is plausible to also verify navigation accuracy at the cortical level before resection. As shown in this large cohort study, vascular cortical structures can be exploited to monitor accuracy, if suitable preoperative vascular data, such as CTA, T1-CE, or ToF angiography, is available at sufficient quality. In 43 (19.2%) out of 224 cases with proper image data, a need for updating neuronavigation by in-plane realignment of vascular representations according to the patient’s anatomy was seen and successfully compensated. As only in-plane misalignment can be accounted for in this way, a careful evaluation across quite a few positions is required to minimize projection errors. In 18 cases, preoperative vascular data was available but not of sufficient quality for vascular alignment purposes due to sparse vascular contrast enhancement or poor image quality. Image quality, especially image resolution and type of acquisition, is one factor influencing accuracy at the level of preoperative planning and patient-to-image registration [7]. In all cases with data being not sufficient for vascular representations, only 2D vascular data with slice thickness above 3 mm or low contrast data was available, not capable of visualizing delicate cortical structures, supporting the need for standardized preoperative high-quality imaging.

In the absence of uniquely identifiable bony landmarks or suitable vascular data prior to surgery, the use of the cortical profile as a further opportunity to assure high accuracy can be seen. In this study, the cortical profile was used as the last option to verify navigation accuracy after dural opening. As the 2D projections of the outlined object representing the cerebrum are rather complex, the microscope video was superimposed on a 3D visualization of the cerebrum and its accuracy was evaluated by moving the focal plane along the optical axis. Only in the few cases with an obvious mismatch, 2D representations were used successfully for compensation. However, as proposed by [4,26], manual segmentation of single sulci in the area of interest instead of the automatically segmented cerebrum might serve similarly to cortical vascular representations for further straightforward evaluation and spatial realignment if needed. To ease and implement a straightforward approach, automatic segmentation of sulci and vascular structures might be worth considering [51,52].

Since its introduction in the 1990s, neuronavigation and, later on, AR-support, have become indispensable tools for a broad variety of cranial and spinal neurosurgical procedures, such as in brain tumor skull base surgery or transsphenoidal approaches [1,2,3,23], supporting intraoperative surgical orientation, precise planning of the surgical approach, and identification of spatial relationship to functional risk structures and thereby contributing to surgical decision-making and further radical resection with in parallel increased patient safety [4,5,6,7,45,46]. However, the potential advantages of the application of neuronavigation and its application safety critically depend on the highly accurate mapping of image data and patient data throughout surgery, which remains a fundamental and multifactorial challenge [1,2,7,8,16,44,47,48]. Whereas some factors, such as the intrinsic accuracy of the navigation system itself, cannot be increased by the user; however, there are relevant factors that can be optimized. Even though registration accuracy is the most prominent and relevant contributing factor prior to intracranial manipulation, accuracy can be tremendously increased using automated image registration techniques. A key question is whether the observed initial registration quality remains high during surgery. As shown by previous studies and supported by this study, various intraoperative factors lead to a continuous decrease in accuracy making it imperative to intermittently verify and, if necessary, to update navigation employing re-registration, spatial realignment, or intraoperative imaging for additional safety.

Even though AR-support is currently widely used in neurosurgical procedures, AR introduces an additional factor contributing to overall accuracy [38]. AR accuracy thereby incorporates the intrinsic accuracy of the implemented AR technique, quite not influenceable by the user itself, and a user-dependent evaluation of AR calibration within the surgical field assuring a high AR application accuracy. In this study, AR application accuracy was routinely evaluated and optimized at first usage of the operating microscope by matching visualized AR marks and reference array marks, increasing AR and navigation accuracy.

Microscope-based AR-support allows for straightforward monitoring of navigation accuracy using uniquely identifiable landmarks and structures, such as bony edges, vascular structures, or cortical profiles, and allowed for a sufficient compensation of navigation inaccuracies prior to resection, provided that a high microscope calibration accuracy is present [38]. However, adjustments made by AR-supported navigation updates are limited to translational and rotational transformations within a 2D plane. Even though accuracy should be evaluated at various positions spread across the accessible area, adaptions are limited to a certain extent, as depth information of alternations for the 3D geometry cannot be compensated with this approach so far. Local translational corrections along the optical axis might be an aspect of further software Nonetheless, microscope-based AR-supported accuracy monitoring and AR-based navigation update can also be applied in subcortical areas, if uniquely identifiable landmarks are available, such as previously reported in aneurysm surgery [16].

Despite the broad and routine use of HUD integration in neurosurgical operating theatres, alternative approaches, such as head-mounted displays (HDM), have been investigated even before HUD, and were first applied in neurosurgery in the 1990s [53]. Especially in the case of surgical procedures without the need for an operating microscope or macroscopic parts of the surgery, e.g., skin incision, HMDs are an interesting alternative. See-through HMDs (e.g., Magic Leap or HoloLens) allow for a direct projection of AR elements into the field of view while providing hands-free interaction and thereof have shown clear benefits over video-see-through systems. However, there is still a poor clinical acceptance of those systems, partially due to, e.g., a lack of performance, perceptual challenges, and registration or occlusion challenges [54]. The reported system accuracy of HMD integrated into a surgical navigation setup was shown to be in the order of 2 to 5 mm or even 5 to 8 mm, not yet sufficient in surgical procedures requiring high accuracy. The opportunity to intermittently evaluate and potentially update navigation as described here might further increase accuracy and support the adoption of HMD AR devices as a valuable tool in surgical applications.

One limitation of this study is its retrospective character. Thereof, in all cases in this study, only those with documented navigation accuracy checks and spatial realignments were considered, in addition to a predefined order of evaluation strategies (bone, vascular, cortex). To fully account for the ability of AR-support to monitor and increase navigation safety, bony landmarks, vascular landmarks, and cortex representations should be considered in all cases to identify interrelations and optimized strategies for evaluation and compensation order. With further software developments that potentially also compensate for initial depth inaccuracies after durotomy might be developed. Similarly, the use of AR-based navigation verification and update might be extended to the whole course of surgery, identifying other suitable deep-seated landmark structures for navigation updates and obtaining high navigation accuracy throughout the surgery.

## 5. Conclusions

High navigational accuracy is a critical prerequisite in navigation-supported neurosurgical applications. However, accuracy is impacted by a broad spectrum of factors and is known to decrease over time. AR-support has been widely integrated in neurosurgical practice, and has shown to allow for intermediate and straightforward monitoring of accuracy by evaluating the spatial relation of image injections and patient’s anatomy investigating bony key landmarks, cortical vascular structures, or the cortical profile prior to resection; it also allows for the correction of spatial misalignments to improve navigation accuracy prior to resection. In this way, AR-support acts as a complementary tool for compensation of decreased navigation accuracy, being a prerequisite for the identification of, e.g., deep-seated lesions, defining resection margins, or assisting in the preservation of functional risk structures, thereby providing additional safety.

## Figures and Tables

**Table 1:** Use of operating microscope and AR to verify and update navigation accuracy.

Accuracy Evaluation	Number of Cases	Sufficient Accuracy	Insufficient Accuracy/Compensation of Inaccuracies	Other
bony landmarks	85	83	2/2	-
cortical vascular structures	242	181	43/43	18 ^1^
cortex representation	40	38	2/2	-

^1^ No sufficient representation of cortical vascular structures could be obtained due to a lack of sufficient 3D data or poor contrast enhancement.

## Data Availability

The data in this study are available on request from the corresponding author. The data are not publicly available due to privacy restrictions.
